# Crystal structure of bis­(tetra­methyl­thio­urea-κ*S*)bis­(thio­cyanato-κ*N*)nickel(II)

**DOI:** 10.1107/S2056989020015121

**Published:** 2020-11-17

**Authors:** Aleksej Jochim, Rastko Radulovic, Inke Jess, Christian Näther

**Affiliations:** a Institut für Anorganische Chemie, Christian-Albrechts-Universität Kiel, Max-Eyth-Str. 2, D-24118 Kiel, Germany

**Keywords:** crystal structure, nickel thio­cyanate, tetra­methyl­thio­urea, discrete complexes, thermal properties

## Abstract

In the course of our investigations regarding transition-metal thio­cyanates with thio­urea derivatives, the title compound, [Ni(NCS)_2_(C_5_H_12_N_2_S)_2_], was obtained. The asymmetric unit consists of one thio­cyanate anion and one tetra­methyl­thio­urea mol­ecule on general positions, as well as one Ni^II^ cation that is located on a twofold rotational axis. In this compound, discrete complexes are formed in which the Ni^II^ cations are surrounded by two *trans*-N-bonding thio­cyanate anions as well as two *trans*-S-bonding tetra­methyl­thio­urea mol­ecules within a distorted square-planar coordination geometry. The discrete complexes are linked by pairs of weak C—H⋯S hydrogen bonds between the thio­cyanate S and one of the tetra­methyl­thio­urea methyl hydrogen atoms into chains along the crystallographic *a*- and *c*-axis directions, which are combined into layers parallel to the *ac* plane. X-ray powder diffraction proves that a pure crystalline phase was obtained and measurements using thermogravimetry and differential thermoanalysis reveal that the compound decomposes at about 408 K, where all tetra­methyl­thio­urea mol­ecules are lost.

## Abstract 2

The title compound, [Ni(NCS)_2_(C_5_H_12_N_2_S)_2_], consists of discrete complexes with a distorted all-*trans* square-planar coordination geometry that are linked by inter­molecular C—H⋯S hydrogen bonds into corrugated layers parallel to the *ac* plane.

## Chemical context   

Many thio­cyanate coordination compounds are reported in the literature, which mostly consist of discrete complexes containing non-bridging N-terminally coordinated thio­cyanate anions, while compounds in which the metal cations are bridged by these anionic ligands are comparatively rare. Despite this fact, a variety of coordination modes can be found for bridging thio­cyanate anions, which leads to metal–thio­cyanate networks with different dimensionalities and topologies (Wöhlert *et al.*, 2014 ▸; Lin, 2008 ▸; Li *et al.*, 2014 ▸; Suckert *et al.*, 2016 ▸). If these compounds contain paramagnetic metal cations, they are of special inter­est, because thio­cyanate anions can mediate magnetic exchange and thus cooperative magnetic phenomena can be expected (Palion-Gazda *et al.*, 2015 ▸; Mekuimemba *et al.*, 2018 ▸; Mousavi *et al.*, 2020 ▸; Rams *et al.*, 2020 ▸; Mautner *et al.*, 2018 ▸). Our inter­est focuses mainly on transition-metal thio­cyanates with the general composition [*M*(NCS)_2_(coligand)_2_]_*n*_ with *M* = Mn^II^, Fe^II^, Co^II^ or Ni^II^ that consist of linear chains, in which the metal cations are connected by pairs of N- and S-bonding thio­cyanate anions into centrosymmetric *M*_2_(NCS)_2_ units, while the remaining sites of the coordination octa­hedron are occupied by neutral coligands forming a coordination environment in which all ligands are *trans* (Wöhlert *et al.*, 2014 ▸; Werner *et al.*, 2014 ▸, 2015 ▸; Prananto *et al.*, 2017 ▸). In this context, it is noted that the Co^II^ compounds are of special inter­est, because either ferromagnetic behavior or a slow relaxation of the magnetization is observed (Werner *et al.*, 2015 ▸; Neumann *et al.*, 2019 ▸; Rams *et al.*, 2017 ▸, 2020 ▸). Besides these chain compounds with an all-*trans* coordination environment, several other isomers with different *cis*–*cis*–*trans* arrangements of the ligands can be found in which either the coligand, the N-bonding or the S-bonding thio­cyanate are *trans*, while the other ligands are *cis* (Maji *et al.*, 2001 ▸; Shi *et al.*, 2007 ▸; Rams *et al.*, 2017 ▸). For most of these compounds, corrugated chains are observed in which the magnetic exchange is low or negligible (Böhme *et al.*, 2020 ▸; Jochim *et al.*, 2018 ▸). In the case of [*M*(NCS)_2_(4-chloro­pyridine)_2_]_*n*_ (*M* = Co, Ni), two isomeric compounds are observed that contain either linear or corrugated chains, which allowed investigations on the influence of the chain geometry on the magnetic behavior, because both compounds contain the same coligand and thus all differences in the magnetic behavior can be attributed to the structural changes (Böhme *et al.*, 2020 ▸; Jochim *et al.*, 2018 ▸).
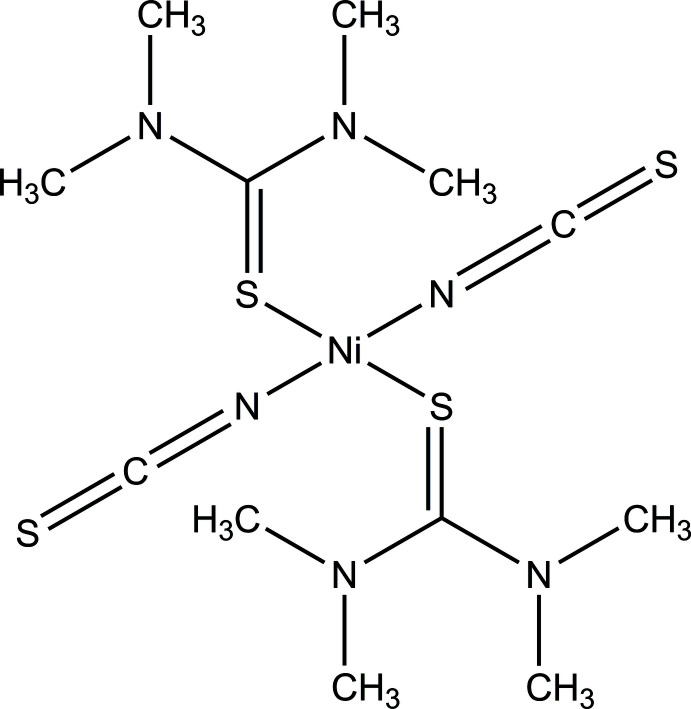


However, to investigate the magnetic properties of trans­ition-metal thio­cyanate compounds in more detail, the influence of the coligands on the structural and magnetic behavior must be investigated systematically. Most of these compounds contain N-donor coligands, whereas compounds with, for example, O- or S-donor coligands are rare (Groom *et al.*, 2016 ▸; Amzel *et al.*, 1969 ▸; Shurdha, *et al.*, 2013 ▸). This is the reason why we became inter­ested in transition-metal thio­cyanate compounds with thio­urea derivatives, where a few compounds have been reported for which either octa­hedral (Amzel *et al.*, 1969 ▸) or tetra­hedral complexes (Jochim *et al.*, 2020*a* ▸,*b* ▸) are observed. Furthermore, some polymeric compounds have been reported in which the metal cations are connected by either the coligands (Nardelli *et al.*, 1966*a* ▸) or the thio­cyanate anions into chains (Nardelli *et al.*, 1966*b* ▸; Jochim *et al.*, 2020*c* ▸). In the course of our systematic investigations we became inter­ested in tetra­methyl­thio­urea as coligand, which upon reaction with nickel thio­cyanate leads to the formation of the title compound [Ni(NCS)_2_(C_5_H_12_N_2_S)_2_] that consists of discrete complexes, in which the thio­cyanate anions are N-terminally coordinated. Phase pure powders of the title compound could easily be obtained, which is confirmed by X-ray powder diffraction (Fig. S1 in the supporting information). The C—N stretching band of the thio­cyanate anion can be found at 2080 cm^−1^, which proves the presence of terminally bonded thio­cyanate anions, in accordance with the results from single crystal X-ray diffraction (Fig. S2). Investigation of the thermal behavior of the title compound shows that it decomposes at about 408 K in one discrete step of 59.0%, which is in agreement with the mass loss calculated for the loss of all coligand mol­ecules of 60.2% (Fig. S3).

## Structural commentary   

**Figure 1:**
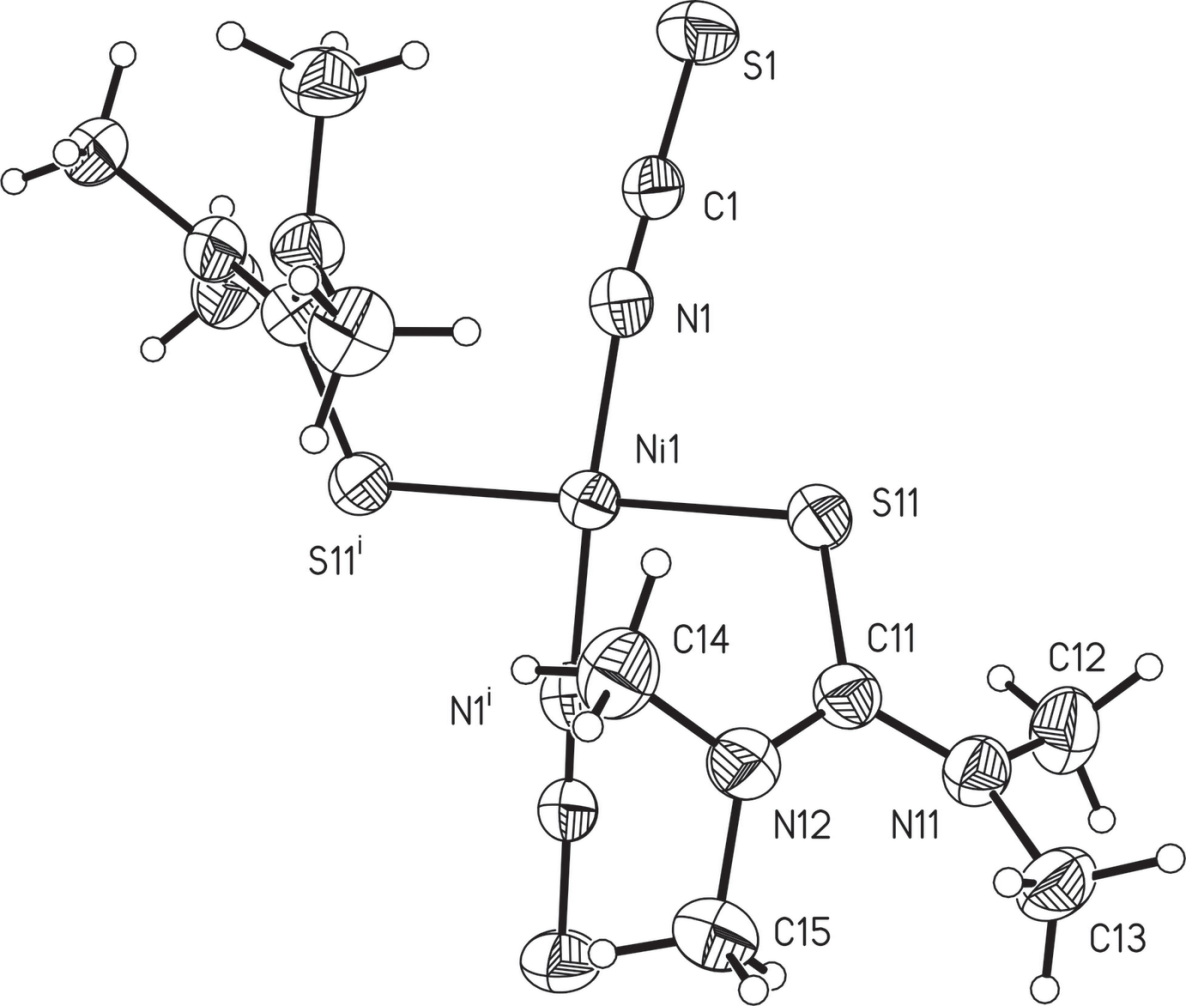
View of the asymmetric unit of the title compound with atom labeling and displacement ellipsoids drawn at the 50% probability level. Symmetry transformations used to generate equivalent atoms: (i) −*x* + 1, *y*, −*z* + 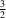.

The asymmetric unit consists of one Ni^II^ cation that is located on a twofold rotational axis as well as one thio­cyanate anion and one tetra­methyl­thio­urea mol­ecule, which both occupy general positions. Each Ni^II^ cation is fourfold coordinated by two *trans* N-binding thio­cyanate anions and two *trans* S-binding tetra­methyl­thio­urea mol­ecules into discrete complexes. (Fig. 1 ▸). The Ni—N bonds are much shorter than the Ni—S bonds and from the angles it is obvious that the Ni cation is in a square-planar coordination geometry (Table 1 ▸). Furthermore, a strong deviation from the ideal angles is found in the coordination environment of the Ni cation, which can probably be attributed to the relatively bulky NMe_2_ groups of the tetra­methyl­thio­urea mol­ecules. This is most pronounced in the N—Ni—N angle, which amounts to 167.47 (16)°. In contrast, for the S—Ni—S angle a smaller deviation with a value of 173.26 (5)° can be found. The tetra­methyl­thio­urea mol­ecules are twisted relative to each other with a C=S⋯S=C torsion angle of 135.0 (2)°. Furthermore, while the thio­urea unit of each tetra­methyl­thio­urea ligand is planar, both NMe_2_ groups are rotated out of this plane by angles of 28.8 (2) and 27.3 (2)°.

## Supra­molecular features   

**Figure 2:**
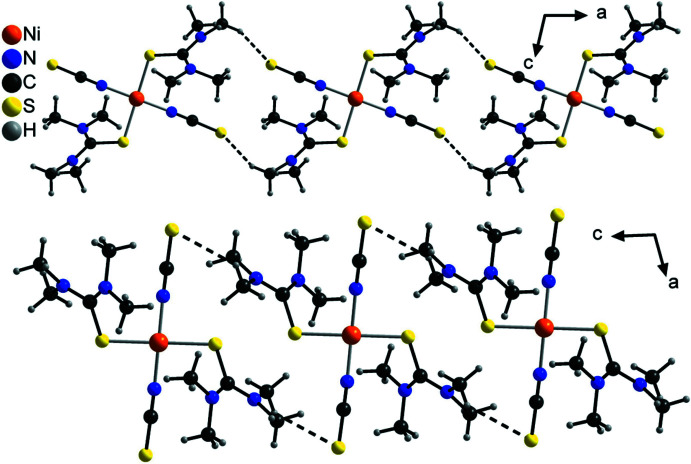
Crystal structure of the title compound with view of the two different chains formed by inter­molecular C—H⋯S hydrogen bonding between S1 and H13*C* (top) and between S1 and H12*B* (bottom). Inter­molecular C—H⋯S hydrogen bonding is shown as dashed lines.

**Figure 3:**
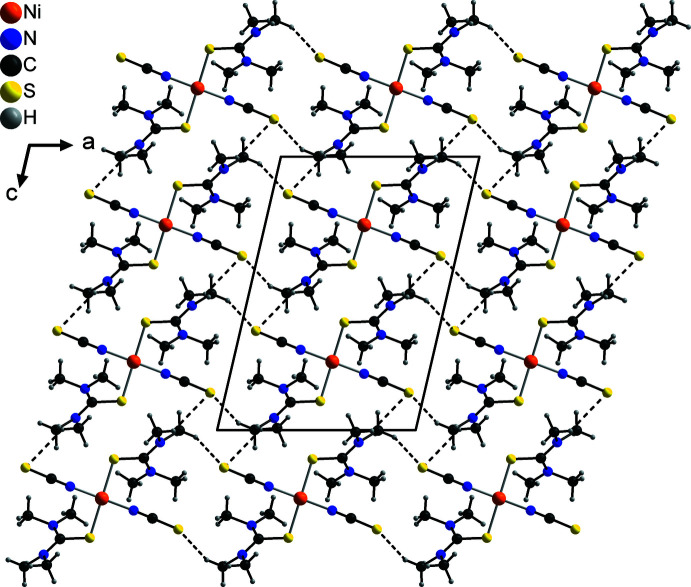
Crystal structure of the title compound with view along the crystallographic *b* axis and inter­molecular C—H⋯S hydrogen bonding shown as dashed lines.

In the crystal structure of the title compound, the discrete complexes are linked by two crystallographically different inter­molecular C—H⋯S hydrogen bonds between the thio­cyanate S atom S1 and the methyl hydrogen atoms H13*C* and H12*C* of the tetra­methyl­thio­urea mol­ecule. In both cases, each two neighbouring complexes are linked into pairs containing 18-membered rings that are located on centers of inversion (Fig. 2 ▸ and Table 2 ▸). These pairs are further linked into chains, which for the hydrogen bonds between S1 and H13*C* proceed along the crystallographic *a*-axis direction and for those between S1 and H12b along the *c*-axis direction (Fig. 2 ▸). These two chains condense into layers parallel to the *ac* plane by centrosymmetric pairs of both crystallographically different C—H⋯S hydrogen bonds (Fig. 3 ▸).

## Database survey   

In the Cambridge Crystallographic Database (CSD, Version 5.41, last update May 2020; Groom *et al.*, 2016 ▸) no transition-metal thio­cyanate compounds with tetra­methyl­thio­urea are reported, but one such compound with cobalt was published recently (Jochim *et al.*, 2020*b* ▸). In this compound, discrete tetra­hedral complexes are found in which the metal cations are coordinated by two N-bonding thio­cyanate anions and two S-bonding tetra­methyl­thio­urea mol­ecules. Several compounds with transition-metal cations and tetra­methyl­thio­urea are reported in the CSD, of which two contain nickel cations. Both consist of discrete binuclear complexes in which the metal cations are connected by thiol­ate ligands. These complexes contain either two Ni^II^ cations with a square-planar coordin­ation geometry (Ito *et al.*, 2009 ▸) or one Ni^II^ and one Fe^II^ cation with square-pyramidal and octa­hedral coordination geom­etries (Ohki *et al.*, 2008 ▸), respectively. Several Ni(NCS)_2_ compounds with other thio­urea derivatives are also found, including polymeric compounds such as [Ni(NCS)_2_(ethyl­ene­thio­urea)_2_]_*n*_ (Nardelli *et al.*, 1966*b* ▸) and discrete complexes like [Ni(NCS)_2_(N,N′-di­ethyl­thio­urea)_4_] (Amzel *et al.*, 1969 ▸), but only one of those contains nickel cations with a square-planar coordination geometry (Leovac *et al.*, 1995 ▸).

## Synthesis and crystallization   

**General**

Ni(NCS)_2_ was synthesized using a procedure described in Jochim *et al.* (2018 ▸). The reagents NiSO_4_·6H_2_O and Ba(NCS)_2_·3H_2_O, which were used for this, were obtained from Merck and Alfa Aesar, respectively.

**Synthesis**

To synthesize a powder sample, a mixture of Ni(NCS)_2_ (0.50 mmol, 87.4 mg) and tetra­methyl­thio­urea (1.00 mmol, 132.2 mg) was stirred in 0.5 mL of ethanol for one day. The black residue was filtered off and washed with *n*-heptane. Single crystals were grown by slow evaporation of the filtrate obtained from a similar reaction. In this case, Ni(NCS)_2_ (0.25 mmol, 43.7 mg) and tetra­methyl­thio­urea (1.00 mmol, 132.3 mg) were reacted in 0.5 mL of *n*-butanol for one day, after which the residue was filtered off. Elemental analysis calculated for C_12_H_24_N_6_NiS_4_ (439.32 g mol^−1^) C 32.81, H 5.51, N 19.13, S 29.20, found: C 32.77, H 5.42, N 19.07, S 29.18. IR (ATR): ν_max_ = 3025 (*w*), 3008 (*w*), 2954 (*w*), 2926 (*w*), 2164 (*w*), 2080 (*s*), 1555 (*s*), 1492 (*m*), 1461 (*m*), 1441 (*m*), 1415 (*w*), 1378 (*s*), 1259 (*m*), 1209 (*w*), 1156 (*s*), 1109 (*s*), 1100 (*s*), 1060 (*m*), 1055 (*m*), 941 (*w*), 878 (*s*), 845 (*s*), 653 (*m*), 612 (*m*), 490 (*m*), 478 (*m*), 468 (*m*), 408 (*m*) cm^−1^.

**Experimental details**

Elemental analysis was performed using an EURO EA elemental analyzer fabricated by EURO VECTOR Instruments. The IR spectrum was measured using an ATI Mattson Genesis Series FTIR Spectrometer, control software: *WINFIRST*, from ATI Mattson. The XRPD measurements were performed with Cu *K*α_1_ radiation (λ = 1.540598 Å) using a Stoe Transmission Powder Diffraction System (STADI P) that is equipped with a MYTHEN 1K detector and a Johansson-type Ge(111) monochromator. DTA–TG measurements were performed in a dynamic nitro­gen atmos­phere (5 NL h^−1^) in Al_2_O_3_ crucibles using a STA-PT 1000 thermobalance from Linseis. The instrument was calibrated using standard reference materials.

## Refinement   

Crystal data, data collection and structure refinement details are summarized in Table 3 ▸. All non-hydrogen atoms were refined with anisotropic displacement parameters. The C-bound H atoms were positioned with idealized geometry (C—H = 0.98 Å) allowing them to rotate, but not to tip and refined isotropically with *U*_iso_(H) = 1.5*U*_eq_(C).

## Supplementary Material

Crystal structure: contains datablock(s) I. DOI: 10.1107/S2056989020015121/tx2033sup1.cif

Structure factors: contains datablock(s) I. DOI: 10.1107/S2056989020015121/tx2033Isup2.hkl

Figure S1. Experimental (top) and calculated (bottom) PXRD pattern of the title compound measured with Cu-radiation. The cell parameters for the calculated pattern were obtained from a Pawley fit for which the parameters obtained from single crystal diffraction were used as initial values. DOI: 10.1107/S2056989020015121/tx2033sup3.tif

Figure S2. IR spectrum of the title compound. Given is the value of the CN stretching vibration of the thiocyanate anions. DOI: 10.1107/S2056989020015121/tx2033sup4.tif

Figure S3. DTG, TG and DTA curve of the title compound measured with 4 C/min. in a dynamic nitrogen atmosphere. DOI: 10.1107/S2056989020015121/tx2033sup5.tif

CCDC reference: 2044233

Additional supporting information:  crystallographic information; 3D view; checkCIF report

## Figures and Tables

**Table 1:** Selected geometric parameters (Å, °)

Ni1—N1	1.844 (3)	Ni1—S11	2.2259 (7)
			
N1—Ni1—N1^i^	167.47 (16)	N1^i^—Ni1—S11	93.93 (8)
N1—Ni1—S11	86.80 (8)	S11^i^—Ni1—S11	173.26 (5)

Symmetry code: (i) 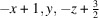.

**Table 2:** Hydrogen-bond geometry (Å, °)

*D*—H⋯*A*	*D*—H	H⋯*A*	*D*⋯*A*	*D*—H⋯*A*
C12—H12*B*⋯S1^ii^	0.98	3.01	3.821 (3)	140
C13—H13*C*⋯S1^iii^	0.98	2.93	3.798 (3)	148

Symmetry codes: (ii) 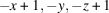; (iii) 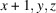.

**Table 3:** Experimental details

Crystal data	
Chemical formula	[Ni(NCS)_2_(C_5_H_12_N_2_S)_2_]
*M*_r_	439.32
Crystal system, space group	Monoclinic, *P*2/*c*
Temperature (K)	200
*a*, *b*, *c* (Å)	10.7245 (3), 6.2050 (3), 15.1579 (5)
β (°)	103.140 (3)
*V* (Å^3^)	982.28 (6)
*Z*	2
Radiation type	Mo *K*α
μ (mm^−1^)	1.42
Crystal size (mm)	0.12 × 0.09 × 0.07

Data collection	
Diffractometer	Stoe IPDS2
Absorption correction	Numerical (*X-RED* and *X-SHAPE*; Stoe & Cie, 2002 ▸)
*T*_min_, *T*_max_	0.716, 0.874
No. of measured, independent and observed [*I* > 2σ(*I*)] reflections	9982, 1945, 1607
*R*_int_	0.070
(sin θ/λ)_max_ (Å^−1^)	0.617

Refinement	
*R*[*F* ^2^ > 2σ(*F* ^2^)], *wR*(*F* ^2^), *S*	0.038, 0.098, 1.05
No. of reflections	1945
No. of parameters	109
H-atom treatment	H-atom parameters constrained
Δρ_max_, Δρ_min_ (e Å^−3^)	0.39, −0.36

Computer programs: *X-AREA* (Stoe & Cie, 2002 ▸), *SHELXS97* (Sheldrick, 2008 ▸), *SHELXL2018/3* (Sheldrick, 2015 ▸), *XP* (Sheldrick, 2008 ▸), *DIAMOND* (Brandenburg & Putz, 1999 ▸) and *publCIF* (Westrip, 2010 ▸).

## supplementary crystallographic information

### Crystal data

**Table:**

[Ni(NCS)_2_(C_5_H_12_N_2_S)_2_]	*F*(000) = 460
*M**_r_* = 439.32	*D*_x_ = 1.485 Mg m^−^^3^
Monoclinic, *P*2/*c*	Mo *K*α radiation, λ = 0.71073 Å
*a* = 10.7245 (3) Å	Cell parameters from 9982 reflections
*b* = 6.2050 (3) Å	θ = 2.0–26.0°
*c* = 15.1579 (5) Å	µ = 1.42 mm^−^^1^
β = 103.140 (3)°	*T* = 200 K
*V* = 982.28 (6) Å^3^	Block, black
*Z* = 2	0.12 × 0.09 × 0.07 mm

### Data collection

**Table:**

Stoe IPDS-2 diffractometer	1607 reflections with *I* > 2σ(*I*)
ω scans	*R*_int_ = 0.070
Absorption correction: numerical (X-Red and X-Shape; Stoe & Cie, 2002)	θ_max_ = 26.0°, θ_min_ = 2.0°
*T*_min_ = 0.716, *T*_max_ = 0.874	*h* = −13→13
9982 measured reflections	*k* = −7→7
1945 independent reflections	*l* = −18→18

### Refinement

**Table:**

Refinement on *F*^2^	0 restraints
Least-squares matrix: full	Hydrogen site location: inferred from neighbouring sites
*R*[*F*^2^ > 2σ(*F*^2^)] = 0.038	H-atom parameters constrained
*wR*(*F*^2^) = 0.098	*w* = 1/[σ^2^(*F*_o_^2^) + (0.056*P*)^2^ + 0.1275*P*] where *P* = (*F*_o_^2^ + 2*F*_c_^2^)/3
*S* = 1.05	(Δ/σ)_max_ < 0.001
1945 reflections	Δρ_max_ = 0.39 e Å^−^^3^
109 parameters	Δρ_min_ = −0.36 e Å^−^^3^

### Special details

**Table:**

Geometry. All esds (except the esd in the dihedral angle between two l.s. planes) are estimated using the full covariance matrix. The cell esds are taken into account individually in the estimation of esds in distances, angles and torsion angles; correlations between esds in cell parameters are only used when they are defined by crystal symmetry. An approximate (isotropic) treatment of cell esds is used for estimating esds involving l.s. planes.

### Fractional atomic coordinates and isotropic or equivalent isotropic displacement parameters (Å^2^)

**Table:**

	*x*	*y*	*z*	*U*_iso_*/*U*_eq_	
Ni1	0.500000	0.29112 (8)	0.750000	0.03467 (16)	
N1	0.3260 (3)	0.2587 (4)	0.70613 (16)	0.0416 (6)	
C1	0.2221 (3)	0.1954 (5)	0.67755 (18)	0.0375 (6)	
S1	0.07891 (8)	0.10731 (15)	0.63716 (6)	0.0536 (2)	
S11	0.51467 (7)	0.31221 (13)	0.60612 (5)	0.0421 (2)	
C11	0.6634 (3)	0.4138 (5)	0.60210 (18)	0.0368 (6)	
N11	0.7271 (2)	0.3274 (4)	0.54463 (16)	0.0402 (5)	
C12	0.7000 (4)	0.1112 (6)	0.5071 (2)	0.0551 (8)	
H12A	0.658143	0.026358	0.546631	0.083*	
H12B	0.780347	0.041141	0.502878	0.083*	
H12C	0.643405	0.121046	0.446582	0.083*	
C13	0.8112 (3)	0.4537 (6)	0.5012 (2)	0.0495 (8)	
H13A	0.801002	0.607219	0.513061	0.074*	
H13B	0.788670	0.427747	0.435733	0.074*	
H13C	0.900282	0.410930	0.525696	0.074*	
N12	0.7143 (2)	0.5845 (4)	0.65156 (16)	0.0410 (5)	
C14	0.6379 (4)	0.7320 (5)	0.6929 (2)	0.0522 (8)	
H14A	0.549871	0.735359	0.656431	0.078*	
H14B	0.674874	0.876929	0.695780	0.078*	
H14C	0.637766	0.682513	0.754302	0.078*	
C15	0.8524 (3)	0.6057 (6)	0.6875 (2)	0.0522 (8)	
H15A	0.894942	0.470956	0.677642	0.078*	
H15B	0.869414	0.636391	0.752599	0.078*	
H15C	0.885381	0.723850	0.656457	0.078*	

### Atomic displacement parameters (Å^2^)

**Table:**

	*U*^11^	*U*^22^	*U*^33^	*U*^12^	*U*^13^	*U*^23^
Ni1	0.0307 (3)	0.0413 (3)	0.0316 (3)	0.000	0.00619 (19)	0.000
N1	0.0423 (15)	0.0489 (14)	0.0335 (12)	0.0005 (11)	0.0085 (10)	0.0004 (10)
C1	0.0364 (16)	0.0446 (15)	0.0319 (13)	0.0021 (12)	0.0086 (11)	0.0017 (11)
S1	0.0354 (4)	0.0637 (5)	0.0597 (5)	−0.0076 (4)	0.0063 (4)	−0.0022 (4)
S11	0.0360 (4)	0.0576 (5)	0.0323 (4)	−0.0074 (3)	0.0069 (3)	−0.0023 (3)
C11	0.0337 (14)	0.0430 (14)	0.0327 (13)	0.0007 (12)	0.0052 (11)	0.0045 (11)
N11	0.0399 (13)	0.0451 (13)	0.0368 (12)	0.0015 (10)	0.0112 (10)	0.0024 (10)
C12	0.066 (2)	0.0556 (19)	0.0474 (18)	−0.0026 (17)	0.0204 (16)	−0.0084 (15)
C13	0.0405 (17)	0.065 (2)	0.0471 (17)	0.0022 (15)	0.0188 (14)	0.0113 (14)
N12	0.0391 (13)	0.0417 (13)	0.0418 (13)	−0.0021 (10)	0.0081 (10)	−0.0029 (10)
C14	0.060 (2)	0.0426 (16)	0.0564 (19)	0.0031 (14)	0.0185 (16)	−0.0051 (14)
C15	0.0418 (17)	0.0580 (19)	0.0539 (18)	−0.0090 (15)	0.0050 (14)	−0.0051 (15)

### Geometric parameters (Å, º)

**Table:**

Ni1—N1	1.844 (3)	C11—N12	1.340 (4)
Ni1—S11	2.2259 (7)	N11—C13	1.460 (4)
N1—C1	1.168 (4)	N11—C12	1.460 (4)
C1—S1	1.614 (3)	N12—C14	1.461 (4)
S11—C11	1.729 (3)	N12—C15	1.464 (4)
C11—N11	1.335 (4)		
			
N1—Ni1—N1^i^	167.47 (16)	N11—C11—S11	119.3 (2)
N1—Ni1—S11	86.80 (8)	N12—C11—S11	122.0 (2)
N1^i^—Ni1—S11	93.93 (8)	C11—N11—C13	122.6 (3)
S11^i^—Ni1—S11	173.26 (5)	C11—N11—C12	122.5 (3)
C1—N1—Ni1	166.5 (3)	C13—N11—C12	113.9 (2)
N1—C1—S1	179.4 (3)	C11—N12—C14	122.6 (3)
C11—S11—Ni1	109.10 (9)	C11—N12—C15	121.9 (3)
N11—C11—N12	118.6 (3)	C14—N12—C15	113.7 (3)

Symmetry code: (i) −*x*+1, *y*, −*z*+3/2.

### Hydrogen-bond geometry (Å, º)

**Table:**

*D*—H···*A*	*D*—H	H···*A*	*D*···*A*	*D*—H···*A*
C12—H12*B*···S1^ii^	0.98	3.01	3.821 (3)	140
C13—H13*C*···S1^iii^	0.98	2.93	3.798 (3)	148

Symmetry codes: (ii) −*x*+1, −*y*, −*z*+1; (iii) *x*+1, *y*, *z*.
